# Hospital and Institutional News

**Published:** 1914-04-04

**Authors:** 


### HOSPITAL AND INSTITUTIONAL NEWS.

### THE LEGISLATION OF APRIL 1.

LAST Wednesday, April 1, the Mental Deficiency Act, the new Poor-Law Institutions and Nursing Orders, and the compulsory notification of ophthalmia neonatorum all came into force. Both the Mental Deficiency Act and the new Orders have been discussed exhaustively in THE HOSPITAL, in the form of interviews and by correspondence, and it only remains here to point out that the new Mental Board of Control, of which Sir William Byrne is chairman, and which has been in existence for five months, has been actively considering how best to carry out the provisions of the new Act. The Royal Commission appointed in 1904 drew attention in its report to the number of persons not requiring careful hospital treatment who were yet housed in mental hospitals, to the punishment of repeated offenders whose condition required treatment rather than correction, and to the large number of mentally-defective persons for whom nothing is done, of both sexes and various grades and degrees of defectivity, who constitute an enormous and miscellaneous class. The Act, therefore, attempts to control the problem presented by these unfortunates by giving power to place them, at the instance of parents or friends or officers of county or borough councils, in an institution. The abuse of such power is guarded against by requiring the certificates of two qualified medical men, and, in the case of those not idiots or imbeciles, by the signature of a judicial authority. The police authority, suspecting any accused person as a defective, may communicate with the local authority, and bring evidence before the court of his or her mental condition. The central authority is the Board of Control, who have powers over the "defectives" and over the administration of the local authorities, who will appoint a committee partly consisting of their own members, partly of experts. The behaviour of these committees will form a criterion of the working of the Act. But the questions remain: "What constitutes mental defect?" and "Who is a Defective?" For this reason our readers will turn with keen interest to the article "Definitions of Mental Deficiency," which appears on page 11.

### WOMEN DOCTORS AND THE LONDON COUNTY COUNCIL.

ON Tuesday at a meeting of the London County Council, presided over by Lord Peel, the names of ten candidates, including three women doctors, were recommended by the Establishment Committee for appointment as full-time medical assistants in the Public Health Department at a salary of £400. As we note elsewhere eight similar appointments were filled last week by promotion. In the case of the women doctors, Miss Pirrell, M.D., Miss Robertson, M.B., and Miss Schwabe, it was stipulated that they should resign their appointments on marriage. On this question an interesting debate followed, in which the Rev. Dr. Scott Lidgett's amendment to omit the ban on marriage was ruled out of order on the ground that under a Standing Order of the Council all women appointed to its service, except teachers and others specially exempted, were required to resign their appointments on marriage. The chairman added that Dr. Scott Lidgett could put himself in order by moving the insertion of a condition that the above ladies should not be required to resign on their marriage. After Mr. E. Ward had pointed out that it was very undesirable in the public interest that they should have among officers performing the same duties, some who were required to resign and some who were not, the debate was adjourned. We cannot believe, however, that matters will be allowed to rest here. The infamous clause in advertisements of some public authorities, saying that applicants for posts must be "without encumbrance," has already been scotched by a discussion in which THE HOSPITAL's readers took a prompt and prominent part. We hope that women doctors and those who have experience of them in public appointments will send us their views without delay. Is the present opportunity of focusing public opinion to be wasted?

### THE MATRON OF BLACKPOOL HOSPITAL RESIGNS.

A STRIKING turn to events has occurred in the deadlock between the medical staff and the board of management of the Victoria Hospital, Blackpool, which was described in our columns last week, by the resignation of the matron. It would, of course, be idle to deny that this will tend to ease the situation. At a meeting of the Blackpool medical men on Monday a reply was drawn up to the latest communication of the board, which unflinchingly stated that until the doctors' demands for the resignation of the matron, and of Dr. Molloy from the board of management, and for the allotment of two seats on the board for the medical staff be complied with, the medical men will not meet the board. To this the board replied that the matron had voluntarily resigned, as she could not be happy in her work under the medical staff. The board went on to say that it had no power to grant seats, as members of the board were elected by donors and subscribers. They also added that they had no power, and did not wish, to call for Dr. Molloy's resignation. How much longer is this institution to be made a spectacle of folly to the hospital world? Is there no governor or subscriber with sufficient public spirit and sense of the importance of hospital matters to come forward and requisition in the proper way an extraordinary meeting, so that an end may be put to the present disastrous farce of a board of management which has estranged the whole of the local profession by appointing a committee to inquire into the existing state off affairs and to issue a report to be discussed at the earliest moment by the subscribers? The matron has set a good example by resigning. The board of management, who have failed to carry on the hospital, could not do better than to follow suit.

### THE MEDICAL SUPERINTENDENTSHIP AT ST. GEORGE'S HOSPITAL.

Two or three months ago, after controversies which readers of The Hospital will remember, the House Committee at St. George's decided to obtain a new medical superintendent in place of Dr. Friend, who resigned last summer. Advertisements of the vacancy were accordingly issued, in which it was laid down that applicants must be practitioners of not less than ten years' standing. We refrained from comment at the time; partly because we desired to avoid even the semblance of putting difficulties in the way of the House Committee over this matter, partly because we thought it possible that a suitable candidate might be found who would fulfil the conditions. It was, however, obvious that the salary offered (£500 a year, with board and residence) was not likely to attract a really first-class man of ten years' professional standing; and we are not surprised to see that after an interval of several weeks the advertisement has been reissued without the clause concerning the ten years' qualification. The announcement now merely specifies that candidates must be not less than twenty-six years of age, and is no longer calculated to exclude the majority of suitable candidates. As now amended there should be no lack of excellent applicants for the vacancy, and we trust that the House Committee will find one who unites the necessary business capacity with the tact and knowledge of the world, as well as the medical experience, which are so necessary for the holder of a post like this.

### A LEEDS ADJUNCT TO HOSPITAL WORK.

The rate of infant mortality in the city of Leeds, which in the south-east district is given as over 150 per 1,000, has led to the formation of what are known as Babies' Welcomes, or centres to which mothers bring their babies for advice and examination at the hands of doctors and trained nurses. The object is to give attention and, if necessary, treatment to the numerous children whose condition or homes are rapidly manufacturing them into hospital cases, but who at the time when the Welcome hopes to catch them are fortunately not ill enough to be taken to hospital. It is essentially constructive and preventive work that has been vigorously undertaken by the Leeds Babies' Welcome Association, which has some 2,000 mothers on its books. The president is Mrs. Kitson Clark, and the extensive work of organising the recent highly successful Welcome Week, during which house-to-house collections reached £785, was undertaken by Mrs. Amyas Longstaffe. The Association at present possesses seven Welcomes, and at the Central Welcome is a nursery where, by a payment of 1s. 9d. per week, mothers can have their babies cared for by day or night in those stages which, unless arrested by more nursing than most working-class mothers can give, will inevitably lead to disease requiring hospital treatment. The nursery contains eight cots, and a trained sister is in charge, with four probationers. Leeds, having awoke to the fact that among the Welcome babies the death-rate has fallen to 30 per cent., is encouraging the new movement; but if it is to grow to the proper proportions at least ten Welcomes are necessary. Surely, while the extension to the Leeds Infirmary is being awaited, Leeds people will make a special effort to ensure the success of this very valuable adjunct to hospital work.

### GIFT OF A HOSPITAL TO ORKNEY.

The Trustees of the Balfour Hospital, Kirkwall, Orkney, have received a letter from Mrs. Garden, widow of the late Bailie Garden, Kirkwall, stating that her family and herself wished "to present a hospital of up-to-date design," and adequate for the county of Orkney, including the burgh of Kirkwall, to Orkney in memory of her late husband. The donors have considered that the Trustees of the Balfour Hospital are the proper body to whom to offer the new hospital, and the latter have gratefully accepted it. The site, building, design, and accommodation are now under consideration. The Balfour Hospital possesses twenty-two beds, ten of which are reserved for fever cases. It also has a horse ambulance attached to it.

### THE ULSTER CHILDREN'S HOSPITAL, BELFAST.

The Most Rev. Dr. Crozier, who presided last week over the annual meeting of the Ulster Hospital for Children and Women, remarked that those who looked back to the very unpretentious days thirty-six years ago were astonished at the development which had taken place undreamt of by the previous generation. As is to be expected in a hospital of this class situated in Ireland both classes of patients, the in and the out, have shown a considerable increase, and although the new hospital only began work between 1911 and 1912 there is already a demand for more beds than it possesses, the number of women's and children's together amounting to the relatively small total of 40. In fact, the number of children treated in the wards has risen in twelve months from 196 to 333. Fortunately, the annual subscription list is still showing a slight improvement, and the amount of work is the best justification for the undertaking of a new hospital three years ago. In the midst of the political excitement which has been centring around Ulster during the past few months it is gratifying to turn to the quiet, steady, unobtrusive work of the Belfast institutions such as this, as an example of the permanent effort put forward, the necessity for which no political activity or change can affect in any degree.

### HOLIDAYS ON THE CONTINENT.

With the approach of Easter the question of holidays arises again, and is one to which institutional workers from the strain of their profession should and must pay great heed. We therefore welcome most cordially a pocket volume called

"Cheap Continental Holidays," just published for a shilling by The Scientific Press, 28 Southampton Street, Strand, if only for the little-known truth which it argues with masterly succinctness that a continental holiday may and can be cheaper than one in England. To stay at Bruges is to learn this lesson in the most delightful way; but a very good substitute is to read Madame Grace Vallois' volume. As Travel Correspondent of *The Nursing Mirror* she has had unrivalled experience of arranging cheap holidays for slender purses; but nurses are not the only readers whom she here has in view, because if a woman can travel abroad for a week or even longer for £5—a really quite possible feat if the directions in this volume be carried out—a man can travel even more cheaply. He needs less luggage, he can be more independent of his surroundings, he can go alone. But where? Fourteen delightful trips are described by the author, and the admirable details and practical advice with which the volume opens should drive many hesitant and would-be travellers abroad this Easter; and if they buy this volume they will have no cause to lament the experiment.

### NEW SECRETARY OF THE BELGRAVE HOSPITAL FOR CHILDREN.

Mr. Cuthbert Panter, of the Great Northern Central Hospital, has been appointed secretary of the Belgrave Hospital for Children in succession to Mr. Thomas W. Gregg, who, as previously announced, has gone to the Bristol General Hospital as secretary on the retirement of Mr. Thomas Thwaites. Mr. Panter has had eleven years of hospital experience, which began for him at St. Mary's Hospital, where for two years he held the post of secretary's clerk. He then became clerk-accountant at the National Hospital for the Paralysed and Epileptic, where he remained for a period of five years. His next appointment was that of assistant secretary at the Great Northern Central Hospital, which, after four years, he now leaves to take up the duties of his new post. The Belgrave Hospital for Children, which possesses some forty beds, has made a feature, under Mr. Gregg, of securing the support of children to the hospital, to which end an organisation, known as the "Childer Chaine," was formed four years ago as an outcome of the Ladies' Association. Each member pays an annual subscription of half-a-crown, and is encouraged by the presentation of badges to add as many fresh links as possible. All new members are elected by the committee, and much work has been done by Mr. T. F. Rider and Miss May Carlos towards extending its usefulness in many directions.

### THE RENOVATED CHESTER INFIRMARY.

We are glad to give a brief account this week of the consummation of the Chester Infirmary renovation scheme, which occurred with the opening by the King last week of the new buildings. We have recorded every important step in its progress since that autumn day in 1909 when Sir Henry Burdett paid a visit to the institution and recorded in the visitors' book the fact that the existing buildings were 150 years old, and that the reconstruction of the institution was imperative. At first the hospital authorities jibbed at the estimated cost, but under the patriotic pressure of Mr. Alderman D. L. Hewitt, then Mayor of Chester, the governors were encouraged to persist, and through his influence the balance that remained from the pageant of the summer of 1910 became the nucleus of a fund "for alteration, rebuilding, or the erection of a new hospital." In January 1911 it was suggested that the infirmary should be reconstructed, and a fillip was given to the movement by an anonymous donor who offered £1,000 provided that the work were begun at once. In September 1912 the Duchess of Westminster laid the memorial stone of the new wings which were being added, which practical step was made possible by the gift of £12,500 from Mr. Albert Wood, of Conway, in the previous part of that year. It was the Albert Wood Wings which the King opened by pressing an electric button last week. Mr. W. T. Lockwood, F.R.I.B.A., is the architect.

### THE ADDITIONS AND THEIR MORAL.

What has virtually happened has been the transformation of the old infirmary into the administration block, the conversion of the old wards, and the building of sanitary towers. To these alterations are added two new wings, each a unit, and a new theatre unit. The pipes are laid beneath the building in a subway; the heating is by central stoves and side radiators; the windows are on the draughtless principle, and were supplied by the C.C. Ventilating Co., of Liverpool; and the floors of teak, except in the corridors, which are of terrazzo. The kitchens have been centralised with their sculleries and store-rooms, and now occupy the site of the former out-patient department. The latter new department has been made separate from the hospital, with, of course, an entrance and exit of its own. The cost of the renovation has been £43,000, of which approximately £38,200 has been subscribed. The people of Chester, Mr. Hewitt, and the Chairman of the board, Mr. J. R. Thomson, have made strenuous efforts which deserve recognition, and now that a new chapter in the infirmary's development is to open we can congratulate the new secretary, Mr. William Rutter, on the encouraging fact that he has not his hands tied by obsolete conditions. The moral of this development is, as we insisted in 1909, that the cry of impossibility for want of money is an excuse that need never be made under the voluntary hospital system. What has been done at Chester could be done at Worcester and Wolverhampton. Will they be awake enough to take this moral home, and to give birth in each place to another Mr. Hewitt to set the ball rolling?

### INSTITUTE OF PATHOLOGY AT MIDDLESEX HOSPITAL.

The first director of the new Institute of Pathology in connection with the Middlesex Hospital, which Sir J. Bland-Sutton's generosity has provided, is Dr. Carl H. Browning, who was appointed last week. It is expected that the buildings will be finished during June, by which time the new director will take up his appointment. Dr. Browning, who graduated at Glasgow in 1903, is at present director of the Laboratory of Clinical Pathology at Glasgow, and lecturer on this subject to Glasgow University.

### LIMITING THE STAY OF SANATORIA PATIENTS.

One of the problems which has been raised by the granting of sanatorium benefit is the question of the duration of a patient's stay. The medical officer of the St. Marylebone Tuberculosis Department is reported to have stated that "there was distinctly apparent an unfortunate tendency to limit the stay of all patients in sanatoria to a definite period." The reasons for this, no doubt, include the necessity of making provision for others in the still numerically limited number of beds. Both the patients and the committees entrusted with sanatorium benefit are interested in the problem, and the Sanatorium Benefit Sub-Committee of the London Insurance Committee presented a report last week which contains the following figures:—936 patients are reported to have remained in sanatoria for more than three months, 109 of whom received institutional treatment exceeding six months, and three for more than a year. The report points out that the average stay is reduced by the number admitted for short periods of observation or instruction. Many patients, it seems, consider themselves possessed of the right to remain for the full period entered on the recommendation, or, if no period be stated, "till cured." The crux of the whole matter is this: Is sanatorium benefit intended to include the necessarily expensive provision for those who are virtually disabled, though whose length of life under sanatorium conditions may be considerable and extend to several years?

### THE POOR-LAW ORDER'S EFFECT ON SALARIES.

The increased work thrown upon workhouse medical officers by the new Poor-Law Institutions Order is leading many of them to reconsider their position seriously. In the Weobley Union, the workhouse medical officer, Dr. Clarke, has decided to resign because he cannot give the time required for the additional work imposed by the Order. Dr. Clarke is also the district medical officer, and the Guardians wished to treat the two offices as one and to require him to resign the district medical officership also. This he declined to do, and the Guardians appealed to the Local Government Board. In their reply the Local Government Board say: "The two offices are separate, and in the absence of any special arrangement at the time of appointment the Board do not consider that on resigning one the officer can be properly asked to resign the other." The Guardians decided to make another appeal to the Board, and to point out that it was impossible to work the offices by two medical officers owing to the fact that, except for Dr. Clarke, no medical man lived within three miles of the workhouse. Pending a final settlement Dr. Clarke has consented to assist the Guardians by continuing to act at the workhouse. It is obvious, however, that in the absence of any special agreement at the time of appointment the Guardians cannot legally or justly treat the two appointments as one, and they will either have to appoint a new workhouse medical officer or will have to make such arrangements with Dr. Clarke as will make it worth his while to continue in his present position.

### HOW TO OBTAIN RADIUM IN POOR-LAW INFIRMARIES.

A proposal made by the Portsmouth Board of Guardians points the way by which the difficulty in obtaining radium for use in Poor-Law infirmaries at a reasonable cost can be overcome at any rate in some instances. £2,000 is being raised by public subscription in Portsmouth for the purchase of radium. The Guardians propose, subject to the consent of the Local Government Board, to subscribe £100 to the fund on condition that the radium purchased is made available for use in the Guardians' institutions. On the basis of their contribution the Guardians would be entitled to the use of the radium for about one day in every three weeks. This would permit of the treatment probably of all the cases under the Guardians' care likely to be benefited by radium. An arrangement could be made, and is certainly highly desirable, whereby the medical officers of the Guardians could have the assistance of the medical experts, to whom the radium will no doubt be entrusted, in the selection of the cases and in the supervision of the treatment. This way out of the difficulty should be noted by Guardians elsewhere.

### INSTITUTIONAL HALF-HOLIDAYS.

The celebration this year of the jubilee of the Saturday half-holiday, in connection with which a banquet is to be held on May 8 to assist in raising £5,000 for the objects of the Early Closing Association, is only the latest example of the fact that practically everything which seems most characteristic of modern life is only about half a century old. The locomotive, the census, the weather reports, the cheap newspaper, the too cheap match are all examples—and yet there was a time well within the memory of those who have reached the age of sixty-five when England did not possess her Saturday half-holiday. There were, of course, no week-ends in those days, and when the Stock Exchange first closed early on Saturdays in 1858 more than one hardened member is reported to have left early only to return disconsolate at the unbearable novelty of having a Saturday half-holiday. On hygienic and health grounds early closing must always appeal to the hospital and institutional world, but by one of the ironies of life institutions themselves cannot close or regulate their hours in the same way as "business houses." Indeed, public holidays frequently add to the work of hospitals through the accidents that occur when a large concourse of people is gathered together.

Hospitals cannot close unless and until microbes and mischances agree together also to suspend their activities on Saturday afternoons, and to take a weekly half-holiday. It is forgetfulness of this and similar facts which makes would-be reformers from time to time press upon hospitals the effects of beneficent factory legislation such as the eight hours' day and allied projects. But experiments have enforced the obvious difficulty, if not impossibility, of carrying them out.

### THE REDUCTION IN THE VAGRANT POPULATION OF LONDON.

We referred last week in a leading article to the success which has attended the policy of centralising the administration of the London casual wards. Point is given to our remarks by the census of homeless poor in London lately taken by the Public Health Committee of the London County Council. It will be remembered that the Local Government Board Order transferring the casual wards to the Metropolitan Asylums Board came into force on March 31, 1912. In that year the London County Council census showed there were 1,033 inmates of the casual wards and 1,203 homeless persons in the street on the night the census was taken. This year the census, taken under similar conditions, revealed that the number of persons in the casual wards had fallen to 335 and the number of the homeless to 540. It is obvious from these figures that improved administration has led not only to a reduction of the inmates of the casual wards but also of the homeless population. It is not easy to say what has become of the vagrants who have thus disappeared from the London census. Many of them have no doubt passed into the ranks of the self-supporting, for the number of vagrants in the whole of England and Wales has been diminishing in recent years, but to nothing like the same extent as in London.

### THE SHORTAGE OF HOSPITAL ACCOMMODATION

Elsewhere in this issue we summarise the interim report of the Fabian Society on the working of the Insurance Act, with special reference to the shortage of voluntary hospital beds, which they estimate to amount only to an average, in round figures, of 1 per 1,000 population. In the *New Statesman* of March 28 there was an article on this shortage which contained the following sentence: "Taking the total population of these (the Home) counties, and all the voluntary hospitals, great and small, of every kind, within their borders, we find that the number of hospital beds comes to slightly less than 2 per 1,000." A cursory glance at the "two classes of institutions which to some extent supplement the voluntary hospital service" is then made by brief allusion to infectious hospitals and the Poor-Law infirmaries. The above view, however, does not represent a fair estimate of the proportion of beds to population actually in use, still less of the number which might be made available by centralisation. While it is technically true to say that Poor-Law infirmaries must admit only the destitute, in practice, as every infirmary superintendent knows, public opinion will not allow anyone to be turned way from a publicly supported hospital.

### A PERFECTED HOSPITAL SYSTEM.

There is felt in fact among large numbers of the population no taint attaching to admission to infirmary wards. As to what might be accomplished by centralisation and co-operation we have only to recall how the number of casuals in London has been reduced by transferring the casual wards from the Boards of Guardians to one central authority—namely, the Metropolitan Asylums Board. In an article in The Hospital of April 5, 1913 (page 13), entitled "From Poor-Law Infirmaries to Public Hospitals," we pointed out that "it would be an enormous gain to everybody concerned if the Local Government Board were to follow up its action in regard to casuals by applying it to every other branch of Poor-Law work. . . There are no fewer than thirty-four Poor-Law infirmaries in Greater London, each under a separate Board of Guardians, a most wasteful and unsatisfactory system, for when one such infirmary may be crowded with patients another may be only partially filled." For the way in which the hospital system may be perfected we would refer our readers to The Hospital of March 22, 1913 (page 681), where the necessary co-operative steps are outlined in detail.

### THE LONDON SCHOOL MEDICAL SERVICE.

The London County Council recently decided that for the purposes of the school medical service London should be divided into four districts, each under the direction of a divisional medical officer. Hitherto three medical assistants have been entrusted with this work, and to fill the vacant position Dr. F. C. Lewis has been appointed at a commencing salary of £500 a year, rising by annual increments of £25 to £600 a year. The Council has also appointed eight new full-time medical assistants at the salary of £400 a year, rising to £500. The successful candidates for these positions were Mr. A. R. Cowell, Mr. H. F. Marris, Dr. R. H. Norman, Dr. W. J. M. Slowan, Mr. A. G. Wells, Mr. L. R. Tosswill, Miss Mabel Russell, and Dr. F. T. H. Wood.

### DEATH AFTER SWALLOWING RADIUM.

An inquest was held at Preston last Saturday on the wife of a local tradesman, who died after an operation to recover a tube of radium which she had inadvertently swallowed. The operation was successful, but death was due to poison as a result of a preparation of atropine and hyoscine with which the patient had previously been injected. In the course of his evidence, Dr. Rayner stated that the patient came for the radium treatment of deafness on March 13. A metal cylinder containing radium and mesothorium was inserted alternatively into each ear and nostril, and it was on an attempt being made to withdraw the cylinder from the left nostril that it was found to have disappeared. A search was made unsuccessfully until $x$-rays revealed its presence in the patient's stomach.

How she had swallowed the cylinder without knowing it the witness said he could not understand. Emetics having failed, an operation was decided on. The operation was successful, but shortly afterwards the patient collapsed, and Dr. Frederick Collinson said that, while he could not think that the preparation or administration were unskilful, the injection caused poisoning in some mysterious way. The jury returned a verdict of death from misadventure due to heart failure and the effects of atropine and hyoscine. There is no doubt that the practice of inserting radium tubes into the canals of the body is attended with a risk which has not yet been sufficiently appreciated to produce a satisfactory means of safeguard.

### PHARMACISTS AND RADIOGRAPHY.

The Public Pharmacists' and Dispensers' Association have held an " open " meeting for members and their friends in the Lecture Theatre at Bloomsbury Square, kindly placed at their disposal by the Council of the Pharmaceutical Society. Mr. T. H. W. Idris, J.P., president of the Association, who was in the chair, supported by many prominent institutional pharmacists, introduced Mr. W. E. Schall, B.Sc. (Lond.), who gave an "*X*-Ray Lecture and Demonstration." The construction of a modern *x*-ray installation was explained and the conditions requisite for the production of *x*-rays. The lecturer dealt with the various kinds of *x*-ray tubes in common use, differences in penetrating power of "hard" and "soft" tubes, methods of applying the tubes for therapeutic purposes, and regeneration of these tubes. Mention was also made of the various forms of interrupter in use—the conversion of the ordinary household electrical supply into a secondary current of high voltage. Incidentally, it was noted that *x*-rays are now largely used in gynæcological practice. We understand that many pharmacists are now acting as *x*-ray operators in the large infirmaries and hospitals under the direction of the medical staff.

### APPEALS BY WIRELESS TELEGRAPHY.

The first institutional appeal by wireless telegraphy was made last Saturday by the orders of the Marconi Company on behalf of the National Institute for the Blind. The orders of the Marconi Company in this connection are of historic interest and therefore worth quoting. They run as follows: "28th March, 1914, operators all long-distance steamers *via* Cape Cod, also *via* Poldhu, offer commanders every ship you meet to-night's appeal for the blind; also ask every ship relay appeal through to ships on every ocean." The appeal records the opening of the new premises of the National Institute for the Blind by the King, and his Majesty's appeal for Braille books, on the need for which we published a letter from Mr. C. Arthur Pearson last week. The appeal proceeds: "May we ask you to arrange during voyage subscriptions to this first appeal on record made by wireless? The appeal is made to all on board British ships and even to sympathetic friends on ships flying other flags who are grateful they are not blind. Kindly send proceeds to Lord Mayor's Fund for the Blind, Mansion House, London. This message sent you gratis by kindness Marconi Company. (Signed) Arthur Pearson, honorary treasurer, National Institute for the Blind, Great Portland Street, W."

### THE DOCTORS' WIVES ASSOCIATION.

Letters and inquiries continue to reach us on the suggested formation of an Association of Doctors' Wives, for the purpose of achieving by co-operation a free service and purchasing agency. We can only repeat in this issue that full particulars may be obtained on application to "Co-operation," c/o The Scientific Press, Limited, 28 Southampton Street, Strand, W.C., when a reprint of the original article which appeared in the Special Number of The Hospital on March 21 will be promptly forwarded. If the necessary number of signatures of doctors' wives is to be obtained before April 18 (a date which was originally fixed to test the possibilities of forming such an Association without delay) it is essential that each conditional member should herself become a centre of interest by discussing the project with all the doctors' wives of her acquaintance who are likely to be in favour of the scheme. Proposals have already been made that women doctors should be eligible for membership, and there is no doubt that with a preliminary membership of 1,000 the Doctors' Wives Association should be able to help the doctor and the doctor's wife in a variety of ways—as was outlined under the respective headings of the original article—and more particularly by enabling her to purchase under expert advice, when required, numerous goods at particularly favourable prices. We reprint on page 18 the coupon which must be signed and filled in by every would-be member of the proposed Association.

### THIS WEEK'S DRUG MARKET.

Interest is maintained in several articles, but, generally speaking, business in the drug market is rather quiet, and is likely to remain so until after the Easter holidays. The unsettled political situation appears to have had little influence upon the market. The results of the fishing in Norway have continued good, but there is little change in the value of cod-liver oil; more business, however, has been done. As a result of a reduction in the price of quicksilver, makers of calomel, corrosive sublimate, and other salts of mercury have reduced their quotations. The price of opium is well maintained. Citric acid is again dearer, and still higher prices are expected; tartaric acid also has an upward tendency in price, and quotations for seidlitz powder and tartarated soda have been raised. The price of santonin is expected to be raised at any moment, notwithstanding the extreme figure already quoted. Menthol is unchanged. American oil of peppermint is dearer. The demand for cocaine continues extremely dull, and prices are unchanged.

